# Verification of tomotherapy dose delivery

**DOI:** 10.4103/0971-6203.54856

**Published:** 2009

**Authors:** S. M. Pelagade, B. R. Paliwal

**Affiliations:** Department of Medical Physics, The Gujarat Cancer & Research Institute, NCH Campus, Asarwa, Ahmedabad-380016, India; 1 Department of Human Oncology, K4/310 Clinical Science Centre, 600 Highland Avenue, University of Wisconsin-Madison, Madison, WI 53792, USA

**Keywords:** Dose, gamma index, tomotherapy

## Abstract

Seventy-one patient-specific delivery quality assurance (DQA) plans for the Tomotherapy HI-ART II helical tomotherapy system (TomoTherapy, Inc., Madison, WI, USA) were measured using film and ion chamber. The agreement in absolute point dose was 1.19 ± 0.79%, 1.91 ± 1.39%, 2.14 ± 1.3%, 1.3 ± 0.73% and 1.67 ± 1.5% for head and neck, prostate, pelvis-abdomen sites, and for all other sites. The spatial agreement between the calculated and the measured film dose distributions was evaluated using the gamma metric distribution. The average frequency versus gamma interval was plotted as a bar graph to quantify the gamma index variation inside the region of interest for each body site.

## Introduction

The Tomotherapy HI-ART II helical tomotherapy system (Tomotherapy, Inc., Madison, WI, USA) achieves highly conformal dose distributions using a helical-CT like IMRT delivery paradigm.[1,2] Daily image-guidance is provided by an integrated megavoltage CT system used to reduce set-up errors allowing more accurate targeting of dose to the intended volume. Intensity modulation is achieved using a binary multi-leaf collimator that has a leaf switching speed of 20 ms providing a large number of intensity levels. This system delivers a highly conformal dose through synchrony of gantry rotation, couch translation, accelerator pulsing and intensity modulation provided by the binary MLC. The complex dynamic delivery requires pre-treatment patient specific delivery quality assurance (DQA) to ensure accurate delivery.[3–5]

IMRT treatment consists of steep dose gradients throughout the treatment volume. Small mis-registrations of these gradients can lead to hot and cold spots in the composite dose. More importantly, a systematic shift in the dose distribution will create a geometrical miss of the target region. Consequently integrating spatial dosimeters such as film, ion chamber arrays and gel dosimeters are used to measure composite dose which is then analyzed using metrics like dose profile comparisons or spatial distributions of distance-to-agreement (DTA) and gamma index (γ). In many cases these spatial dosimeters are normalized to a point that is calibrated to absolute dose using a point ion chamber measurement. The DTA is the distance between a measured dose point and the nearest point in the calculated distribution with the same dose value. The second methodology includes absolute dose variation (ΔD) at the points of choice between the calculated and measured doses. These two methodologies were combined, with the low gradient regions evaluated with respect to absolute dose variation (ΔD) and the high gradient regions evaluated by a distance-to-agreement (DTA) value (Δd). Low, *et al,* developed a technique to simultaneously incorporate both DD and DTA values into the quality index called gamma.[6] The gamma index shows the difference between the calculated and measured doses relative to the acceptance tolerances. It represents disagreement in the regions that fail the acceptance criteria and indicates quality in the regions that pass.

The acceptance criteria for the film evaluation using gamma index, varies from user to user. For example, Winkler *et al*. (cite) considered a dose difference criteria of ΔD=5 % and DTA criteria of Dd=3 mm and VanDyk *et al*. (cite) used 3%/3mm. We used the VanDyk criteria in our analysis.[7]

In this investigation, we reviewed the patient specific delivery quality assurance for the patients of Tomotherapy HI-ART II helical tomotherapy system. We evaluated the agreement between the planned and delivered point dose measurements are made with an ion chamber. The agreement between the calculated and measured film dose distributions were evaluated with gamma index calculated for criteria (3%/3mm) as recommended in literature.

## Materials and Methods

**Figure 1:**
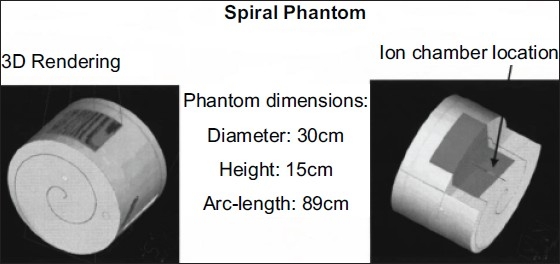
The left hand side image shows 3D rendering of the phantom. The right hand side image shows cross-section view that exposes the location of the ion chamber

In this study we analyzed DQA verification plans for 71 patients (10 cases for head and neck, 18 for prostate, 12 for lung, six for pelvis-abdomen and 25 for “others”) treated using the Tomotherapy HI-ART II system. The patients were categorized according to their disease sites: head and neck, prostate, lung, pelvis-abdomen and “others”. Measurements were recorded using spiral phantom as shown in Figure 1.

The solid water cylindrical phantom of 30 cm diameter and 15 cm height with a machined spiral cavity for film placement.[8] The arc length of the spiral cavity is 89 cm. The phantom was also machined in several locations to place an ionization chamber to verify the delivered dose at these points. The advantage of using this phantom is that the radiation beam is always perpendicular to the film.

Kodak XV film is cut to fit the spiral (5.5 cm × 17 cm) and slide into the spiral. These pieces are then taped at the sides at the joining ends to avoid slippage in the phantom. Straps are placed around the circumference of the phantom and tightened; this constricts the spiral and helps to eliminate air gaps as far as possible. The phantom is then encased in a rounded housing of Lucite™ for additional build up and to allow the use of non-coplanar fields.

The spiral phantom was aligned using the DQA module of the tomotherapy planning station software using the patient positioning red lasers. A patient specific DQA plan was created for each case and delivered to the DQA spiral phantom. Point doses were sampled from the plan at the location where ion chamber measurements were acquired. An ion chamber (NAC mini ion chamber) was placed in the appropriate hole. A treatment was delivered and the detector response was recorded. (Ideally, the isocenter dose value would be measured and compared with the calculated value by treatment planning system) The percentage dose difference between the calculated dose, Dc, and the measured dose, Dm at a specific depth was given by (Dc-Dm) × 100% / Dm.[9]

After irradiation, the film was left to stabilize for the same period of time as its corresponding calibration film before processing. The films were processed using a Kodak RP X-OMAT processor. The treatment film was then scanned at 0.36×0.36 mm^2^ resolution using a VIDAR VXR-16 Dosimetry Pro scanner (VIDAR Systems Corporation, Herndon, VA). The digitized optical density map was then converted to a dose map with the sensitometric curve using MATLAB. All films came from the same film batch and were processed under the same conditions. Since we are interested in relative rather than absolute dosimetry, the change in processor conditions from the time the calibration curve was created to when the spiral phantom films were developed is not considered.

The calculated dose distribution corresponding to the plane of the measured dose distribution was then determined according to the position of the spiral phantom using Tomotherapy Inc. software. The calculated dose distribution was then upsampled using nearest neighbor interpolation such that the dose pixel size of the measured and calculated dose distributions were both 0.36 × 0.36 mm^2^.

The film dose distribution is compared to the calculated dose distribution using the DQA Analysis Tools provided on the TomoTherapy Planner workstation. The DQA Analysis Tool allows one to register the film data to the calculated dose and then extract 1D dose profiles, as well compare the 2D distributions using isodose lines and the gamma distribution.

## Results and Discussions

**Figure 2:**
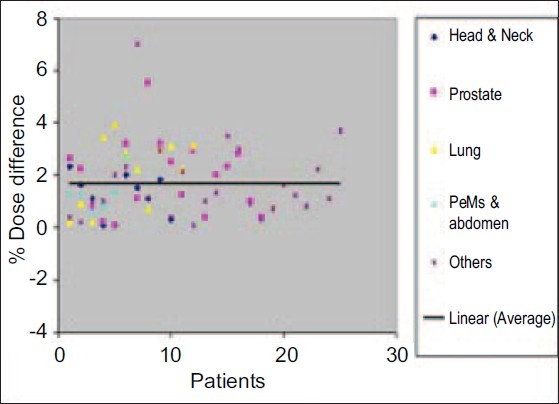
% Dose difference (absolute) variation for all 71 Cases

The absolute percentage difference for the point dose measurements for different body sites is plotted in Figure 2. The average percentage dose difference and standard deviation for the set of cases evaluated for each body site were: 1.19±0.79% for head and neck, 1.91±1.39% for prostate, 2.14±1.3% for lung, 1.3±0.73% for abdomen and pelvis and 1.67±1.5% for other sites. The much larger standard deviation is noted for prostate, lung and other sites. This may be due to dose gradient at that point.

**Figure 3:**
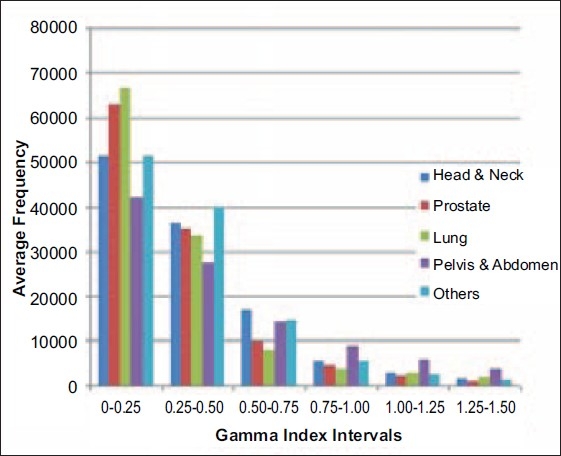
Gamma Analysis: On Y-axis average frequency represents the average dose pixels falling in one of the eight gamma index interval. The bar graph provides the average frequency value (for all 10 patients of each site) for various gamma intervals 0-0.25, 0.25-0.50, 0.50-0.75, 0.75-1.00, 1.00-1.25 and 1.25-1.50 from frequency gamma histogram

The DQA Analysis Tool provides a frequency histogram of the 2D distribution of gamma values, where frequency represents the number of dose pixels whose values fall within an interval. Ten patients per site were considered in this study. The average frequency for each gamma index interval (0-0.25, 0.25-0.50, 0.50-0.75, 0.75-1.00, 1.00-1.25 and 1.25-1.50) was noted for each patient. The average of frequency for each gamma index interval for ten patients was plotted against gamma index interval as a bar graph as shown in Figure 3.

Δ*d* is the distance to agreement criteria and Δ*D* is the dose difference criteria. Gamma was calculated for Δ*d=3 mm* and Δ*D=3 %*. γ=1 describes the surface of a volume of acceptance for the given criteria of Δ*d* and Δ*D*. Any calculated dose pixel where γ is les than or equal to one has met the acceptance criteria and a calculated dose pixel where γ is greater than one has failed the acceptance criteria.

## Conclusion

In this paper we reviewed patient-specific quality assurance associated with an exciting new technology which recently became commercially available. In few cases it is found that the absolute dose difference is up to four per cent. This indicates the need for patient-specific QA procedures. The causes of observed differences in dose calculation is still under investigation.
